# Modeling and simulation of diffusion and reaction processes during the staining of tissue sections on slides

**DOI:** 10.1007/s00418-022-02118-9

**Published:** 2022-06-06

**Authors:** Johannes D. M. Menning, Thomas Wallmersperger, Matthias Meinhardt, Adrian Ehrenhofer

**Affiliations:** 1 Technische Universität Dresden, Institute of Solid Mechanics, George-Bähr-Straße 3c, 01069 Dresden, Germany; 2 University Hospital Carl Gustav Carus Dresden, Institute of Pathology, Fetscherstraße 74, 01307 Dresden, Germany; 3 Technische Universität Dresden, Dresden Center for Intelligent Materials, School of Engineering Sciences, George-Bähr-Straße 3c, 01069 Dresden, Germany

**Keywords:** Histological staining, Finite element simulation, Numerical simulation, Reaction–diffusion equation, Image segmentation, Beer–Lambert law

## Abstract

Histological slides are an important tool in the diagnosis of tumors as well as of other diseases that affect cell shapes and distributions. Until now, the research concerning an optimal staining time has been mainly done empirically. In experimental investigations, it is often not possible to stain an already-stained slide with another stain to receive further information. To overcome these challenges, in the present paper a continuum-based model was developed for conducting a virtual (re-)staining of a scanned histological slide. This model is capable of simulating the staining of cell nuclei with the dye hematoxylin (C.I. 75,290). The transport and binding of the dye are modeled (i) along with the resulting RGB intensities (ii). For (i), a coupled diffusion–reaction equation is used and for (ii) Beer–Lambert’s law. For the spatial discretization an approach based on the finite element method (FEM) is used and for the time discretization a finite difference method (FDM). For the validation of the proposed model, frozen sections from human liver biopsies stained with hemalum were used. The staining times were varied so that the development of the staining intensity could be observed over time. The results show that the model is capable of predicting the staining process. The model can therefore be used to perform a virtual (re-)staining of a histological sample. This allows a change of the staining parameters without the need of acquiring an additional sample. The virtual standardization of the staining is the first step towards universal cross-site comparability of histological slides.

**Supplementary Information:**

The online version contains supplementary material available at 10.1007/s00418-022-02118-9.

## Introduction

Cancer is one of the leading causes of death worldwide today (Ferlay et al. 2020). In order to discover and treat cancer appropriately, it is necessary to examine the histological characteristics of tumor cells (Jones et al. 2015). The two main ways to diagnose the presence of cancer and its characteristics, such as the degree of malignancy are (i) the staining of histological sections (Falk et al. 2018; Giuliano et al. 2011; Hiddemann and Bartram 2010; Veuthey et al. 2014) and (ii) radiological techniques, e.g., magnetic resonance imaging (Dong et al. 2017). It is anticipated that pathologists will receive support in the diagnosis of diseases from models based on artificial intelligence methods (Ari and Hanbay 2018; Dong et al. 2017; Kather et al. 2019; Sharma et al. 2014). Additionally, methods without the need of preparing classical histological slides were developed (Hollon et al. 2020).

**Fig. 1:**
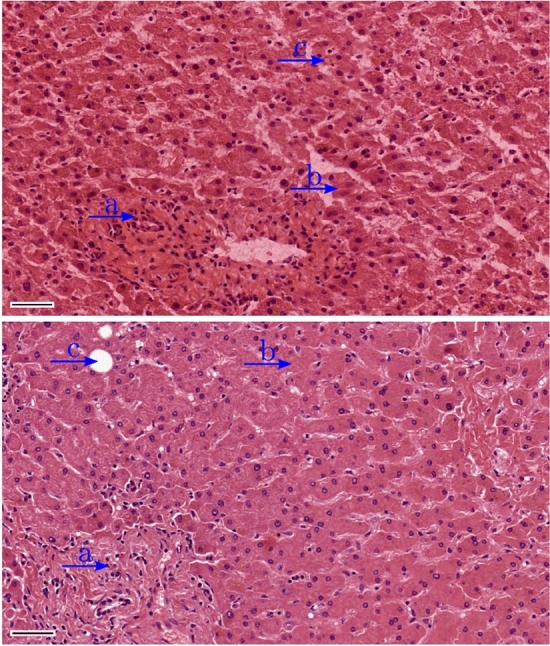
Comparison of a frozen section (*top*) with a paraffin section (*bottom*) from the same patient sample. The region denoted by **a** is a portal field with bile ducts and lymphocytes, **b** denotes liver cells which are arranged in cell strands (trabeculae) and **c** is a fat vacuole. The* scale bar* is 50 µm

The goal of staining is to create a visual distinction between the components of the tissue (see Fig. 1). To obtain a general overview, the cell nuclei and cytoplasm are stained with an appropriate dye.

The most common tissue stains for that purpose are hematoxylin (stains mainly the nuclei) and eosin (stains mainly the cytoplasm) (Lang 2006; Mulisch and Welsch 2010; Suvarna et al. 2019; Veuthey et al. 2014).

The aim of the present paper is to model and simulate the nuclei staining of histological sections with the dye hematoxylin. A histological slide, which was already stained with dye A, could therefore be virtually re-stained with a different dye B. Such a model is feasible because each cell in an organism can be characterized by the structure of its nuclei and cytoplasm (Mulisch and Welsch 2010). Additionally, the dye reacts always in the same way with the respective tissue (Lang 2006).

Modeling and simulation of dyeing in general has been studied mainly with respect to textile fiber dyeing (de Souza et al. 2007; Lin 1992; Reddy et al. 1995). Reddy et al. (1995) used the Langmuir model and compared that to a diffusion equation. de Souza et al. (2007) and Lin (1992) used coupled diffusion–reaction equations. There are also examples like by Winzek and Baumgärtel (1988) who simulated the staining of histological slides or by Irion et al. (1993) who modeled the staining of single-cell organelles.

In the current work, the mechanics of the staining of histological sections is investigated via the methods of building a simplified model and conducting numerical simulations. After a short introduction into the basics of histological staining, the model to describe the diffusion and reaction processes during the staining of histological sections is presented. This model allows the determination of the resulting stain for each nucleus individually, since it is important for an investigator to look at the entire tissue section and not just individual nuclei. Therefore, a workflow was created to derive a two-dimensional representation of the geometry of the scanned histological slide. This geometry is then used to create a finite element mesh for the sample. This procedure is described in the following section, together with the discretization of the problem and the applied initial conditions and boundary conditions. The proposed model is then compared to real histological slides that were created at the Institute of Pathology at the University Hospital Carl Gustav Carus Dresden.

## Materials and methods

### Theoretical framework and continuum-based model

Here, we give a brief introduction to the dye used for the experiments and simulations and how it binds to the tissue sections. Afterwards, the proposed model for the computation of the dye concentration and the resulting color is described. The model is based on continuum mechanics, i.e., the local concentration of the dye changes due to transport that is caused by diffusion.

### Hematoxylin

Hematoxylin is a commonly used dye to stain nuclei (Prentø 2001) and is often used in a combination with eosin (Mulisch and Welsch 2010; Suvarna et al. 2019) but also as a counter stain for immunohistochemical staining (de Cea and Nie 2018; Suvarna et al. 2019). For this reason, the staining of a histological section with hematoxylin was modeled.

The actual dye is not hematoxylin but a combination of the oxidized hematoxylin (called hematein) and a mordant with additional components. There are diverse recipes with varying amounts of hematein, mordant, and other components (Prentø 2001, 2009). In our investigation, Mayer’s hemalum was used for the experimental tissue sections as well as for the numerical simulation.

### Physico-chemical binding theory of tissue staining

Several theories exist that describe the processes involved in tissue staining. The most commonly used theory is the physical–chemical bonding theory, which is based on electrostatic attraction (Mulisch and Welsch 2010; Veuthey et al. 2014). It is assumed that the dye and the tissue have an acidic or basic pH value, which results in a negative or positive charge. After the dye has interacted with the tissue due to the electrostatic attraction, a chemical bond is formed (Mulisch and Welsch 2010; Prentø 2001, 2009; Veuthey et al. 2014). Please note that a dye must have complementary properties to the tissue in order to be able to form a chemical bond. This is necessary in order to compete against other molecules (Prentø 2001).

### Histological changes of tumors

For tumor cells, a de-differentiation occurs, which is accompanied by changes in shape of the nucleus that becomes often enlarged and irregularly shaped and is hyperchromatic. This is also referred to as pleomorphism. In addition, the cytoplasm of the tumor cell may exhibit a staining different from normal cells.

### Transport and binding of the dye

**Fig. 2:**
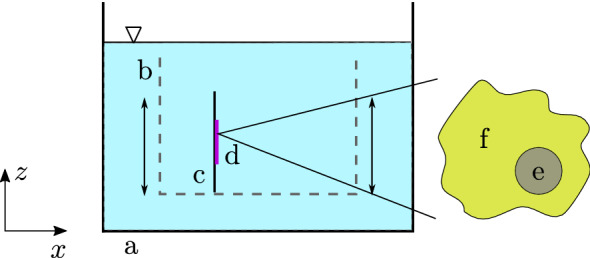
Illustration of a dye basin (**a**) in which the slide rack (**b**) is submerged. A histological slide (**c**) with a tissue sample (**d**) represents multiple histological sections situated on the slide rack. A zoom of this tissue sample shows a simplified model of a cell used in the current work. In this simplification, the cell consists of only two components, the cell nucleus (**e**) and the cytoplasm (**f**)

In literature concerning the mechanics of staining, the diffusion is described to be the dominant factor for the transport of the dye (Goldstein 1980; Irion et al. 1993; Siedel and Zimmermann 1995; Winzek et al. 1987). Therefore, a diffusion term is used to model the transport of the dye. Inside the cell, no difference regarding the diffusion coefficient is made between its single components, e.g., the membranes, cytoplasm, or nucleus. As a consequence, the diffusion in the cell can be modeled as a single step. For a better understanding of the staining process, a dye basin with a histological slide as well as a simplified model of a cell is depicted in Fig. 2. Usually, the glass slides with the attached tissue sections are moved inside the dye basin. Thus, the dye solution is always in motion and is getting mixed. This leads to a constant dye concentration at the surface of the histological slide. Therefore, diffusion is modeled only in the tissue and not also inside the dye basin.

Once the dye has reached its binding sites due to the electrostatic attraction, it forms a chemical bond with the respective tissue (see “Physico-chemical binding theory of tissue staining”).

The binding of the dye to the tissue is a reversible reaction (Ferus-Comelo 2002; Winzek and Baumgärtel 1988). Therefore, the binding of the dye can be represented as a pseudochemical reaction

1 $$A + B \leftrightarrow C$$

where *A* is the free, unbound dye concentration, *B* the tissue with which the dye can form a bond, and *C* is the dye which is bound to the tissue.

The staining can be considered to be a two-step process (Prentø 2009). The first step is the diffusion of the dye into the tissue section and the second step is the binding of the dye to the tissue components. For the simulation of both steps, a diffusion–reaction equation and a reaction equation are fully coupled to form the resulting system of equations. The detailed derivation can be found in Menning (2021).

2 $$\frac{\partial {c}_{\text{f}}}{\partial t}=D{\nabla }^{2}{c}_{\text{f}}-{k}_{\text{on}}{c}_{\text{f}}\hspace{0.17em}\left({c}_{\text{b}}^{\text{max}}-{c}_{\text{b}}\right)+{k}_{\text{off}}\hspace{0.17em}{c}_{\text{b}}$$

3 $$\frac{\partial {c}_{\text{b}}}{\partial t}={k}_{\text{on}}{c}_{\text{f}}\left({c}_{\text{b}}^{\text{max}}-{c}_{\text{b}}\right)-{k}_{\text{off}}{c}_{\text{b}}$$

The used material parameters are the diffusion coefficient *D*, the rate constant which determines the binding rate ${k}_{\mathrm{on}}$, the rate constant which determines the dye release rate ${k}_{\mathrm{off}}$, and the maximal concentration of dye ${c}_{\mathrm{b}}^{\mathrm{max}}$ that the respective part of the cell, in this case the nucleus, can bind. The general variables in Eq. (1) can now be defined with the used variables

4 $${c}_{f}+ \left({c}_{b}^{max} - {c}_{b}\right)\rightleftharpoons {c}_{b}$$

Please note that Eq. (4) is not an exact chemical formula; it is only for better representation. The unknown functions in Eqs. (2) and (3) are the concentration of the unbound dye ${c}_{\mathrm{f}}$ (which is free to move) and the concentration of the bound dye ${c}_{\mathrm{b}}$. The latter describes the concentration of the dye which has formed a chemical bond with the tissue. Therefore, ${c}_{\mathrm{b}}$ is no longer able to diffuse and thus no diffusion term is needed in Eq. (3). Additionally, to the diffusion in the first step (Prentø 2009) describes an ion exchange. This ion exchange is not explicitly modeled. It determines how and where the dye is able to bind to the tissue. Through the common use of Mayer’s hemalum, it is already known that the dye can bind to almost all nuclear acids (Prentø 2001). Equations  (2) and (3) are used to model the transport and the binding of a dye to a histological section. Equations (2) and (3) are similar to the equations used by Lin (1992) to model the staining of textile fibers.

### RGB color model

**Fig. 3:**
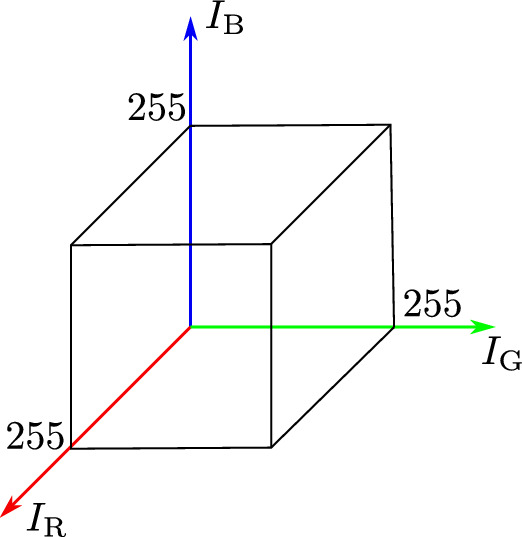
Representation of the color space defined by the red, green, and blue intensity according to Van der Laak et al. (2000)

There exist different models to mathematically describe a color (Bejnordi et al. 2015; Ruifrok et al. 2001; Van der Laak et al. 2000). The model used in this paper is the RGB model, which is an additive color model (Heid and Reith 2010). The three intensities red (R), green (G), and blue (B) span a vector space whose axes are the three intensities (Fig. 3). Typically, the values range between 0 to 1 or from 0 to 255. The latter range was used in the current work. A combination of (0*,*0*,*0) represents the color black and if each intensity is maximal, i.e., 255, the color white is represented (Van der Laak et al. 2000). That is, the lower the intensities, the darker the color.

### Derivation of a color from the dye concentration

Using the equations given in “Transport and binding of the dye”, it is possible to calculate the concentration of the dye bound to the tissue ${c}_{\mathrm{b}}$. To derive the resulting color from this concentration, Beer–Lambert’s law.

5 $$I={I}_{0} \, \mathrm{exp}\left(-\varepsilon {c}_{\mathrm{b}} h\right)$$

is used. It links the intensity of the light after passing the slide *I* with the concentration of the bound dye ${c}_{\mathrm{b}}$ via the extinction coefficient *ε*, the thickness of the slide *h* and the intensity of the light before it enters the specimen *I*_0_ (Bettinger and Zimmermann 1991; Gavrilovic et al. 2013; Ruifrok et al. 2001). The extinction coefficient *ε* is a material parameter of the dye. It determines which wavelengths *λ* of the light are absorbed by the dye and are thus no longer present in the light leaving the slide. Please note that the Beer–Lambert’s law is only valid for dyes that absorb the light like hematoxylin or eosin. Beer–Lambert’s law cannot be applied for dyes that reflect light (Gavrilovic et al. 2013).

The intensities *I* and *I*_0_ as well as the extinction coefficient *ε* are dependent on the wavelength *λ*. The thickness *h* of the slide is a parameter of the slide’s production process. Following Van der Laak et al. (2000), Eq. (5) can be formulated individually for the three colors red (R), green (G), and blue (B)

6 $${I}_{R}={I}_{0,R} \, \mathrm{exp}\left(-{\varepsilon }_{R} {c}_{\mathrm{b}} h\right)$$

7 $${I}_{G}={I}_{0,G} \, \mathrm{exp}\left(-{\varepsilon }_{G} {c}_{\mathrm{b}} h\right)$$

8 $${I}_{B}={I}_{0,B} \, \mathrm{exp}\left(-{\varepsilon }_{B} {c}_{\mathrm{b}} h\right)$$

Equations (6)–(8) can be used to calculate the intensities ${I}_{R}$, ${I}_{G}$ and ${I}_{B}$ and required to derive a resulting color. The extinction coefficient *ε* can be (i) determined independently by experimental investigations, (ii) taken from the literature or be (iii) calculated from data of stained slides, as has been done in the present work.

### Numerical implementation

For the implementation of the introduced equations, the open-source software FEniCS (Alnæs et al. 2015) was used. After the discretization, the initial conditions and the boundary conditions are given in “Initial conditions and boundary conditions”. After that, the used material parameters, are determined in “Material parameters”. In “Finite element mesh from a scanned slide”, the workflow for deriving a FE mesh based on a scanned slide is described.

### Discretization

**Fig. 4:**
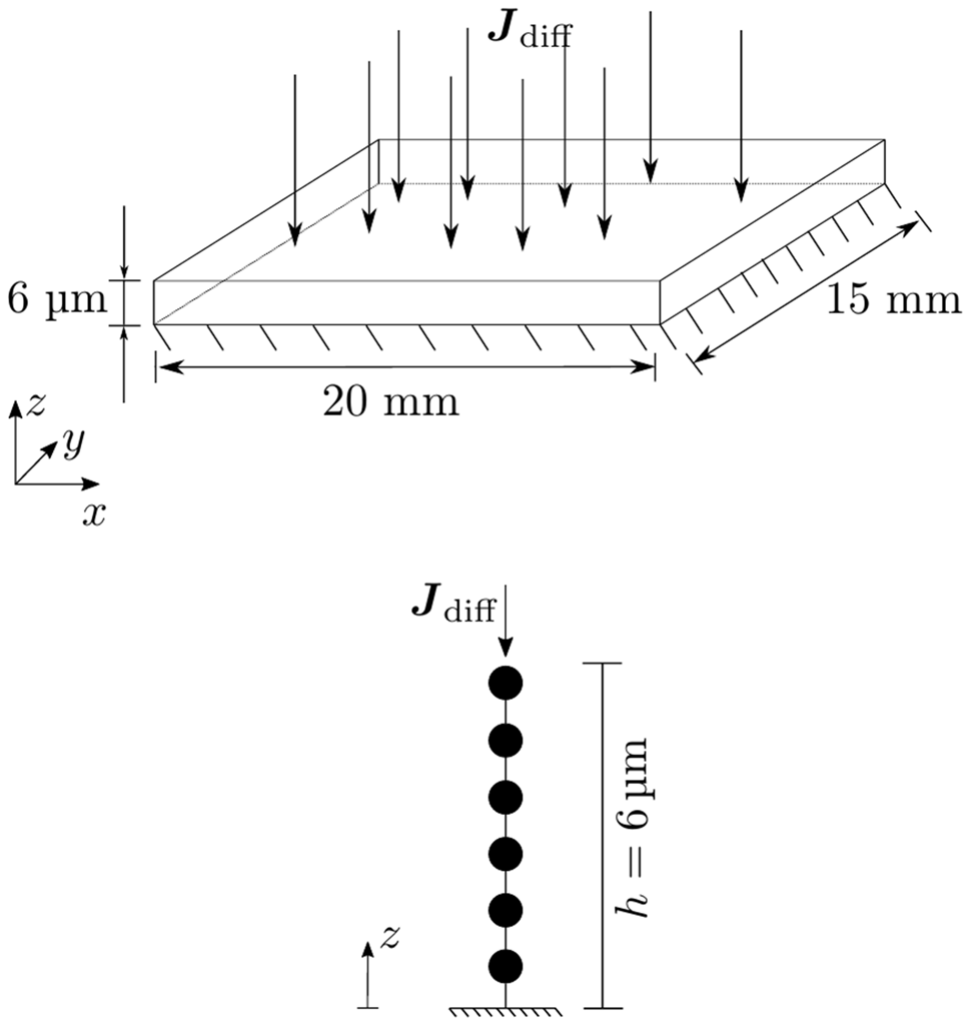
*Top panel*: Schematic representation of a histological section with the diffusional flux ${{\varvec{J}}}_{\mathrm{diff}}$. At the bottom, the tissue is situated on a glass slide which blocks the transport of the dye, represented by the* hatching*.* Bottom panel*: Representation of the discrete one-dimensional FE mesh used to calculate dye transport

The finite element method is used to discretize and solve Eqs. (2) and (3). The required weak formulations are

9 $${\int }_{\Omega }{\dot{c}}_{\mathrm{f}} \delta {c}_{\mathrm{f}} \mathrm{dV}= {\int }_{\partial\Omega }{\varvec{n}}\cdot (D\nabla {c}_{\mathrm{f}}\delta {c}_{\mathrm{f}}) \mathrm{d}A-{\int }_{\Omega }D\nabla {c}_{\mathrm{f}}\cdot \nabla \delta {c}_{\mathrm{f}}\mathrm{ d}V- {\int }_{\Omega }{k}_{\mathrm{on}}{c}_{\mathrm{f}}\left({c}_{\mathrm{b}}^{\mathrm{max}}-{c}_{\mathrm{b}}\right)\delta {c}_{\mathrm{f}} \mathrm{d}V+{\int }_{\Omega }{k}_{\mathrm{off}}{c}_{\mathrm{b}}\delta {c}_{\mathrm{f}} \mathrm{dV}$$

10 $${\int }_{\Omega }{\dot{c}}_{\mathrm{b}} \delta {c}_{\mathrm{b}} \mathrm{dV}= {\int }_{\Omega }{k}_{\mathrm{on}}{c}_{\mathrm{f}}\left({c}_{\mathrm{b}}^{\mathrm{max}}-{c}_{\mathrm{b}}\right)\delta {c}_{\mathrm{b}} \mathrm{d}V-{\int }_{\Omega }{k}_{\mathrm{off}}{c}_{\mathrm{b}}\delta {c}_{\mathrm{b}} \mathrm{dV}$$

with the two weighting functions ${\delta c}_{\mathrm{f}}$ and ${\delta c}_{\mathrm{b}}$ and the integrals over the area *A* and the volume *V*. **n** represents the normal vector at the boundary in outwards direction. Typical dimensions of a histological section are 20 mm × 15 mm in the plane (Mulisch and Welsch 2010) and only a few micrometer in thickness. For the sections actually used in the experiments, a cutting thickness of $h=6 \mathrm{\mu m}$ was set at the microtome. Due to these striking differences in dimensions, diffusion over *h*, i.e., along the *z*-axis (Fig. 4 (top)) will be much faster than in the *xy*-plane. Therefore, the assumption was made that diffusion in the *xy*-plane can be neglected. Thus, the problem is considered to be *one-dimensional* in *z*-direction. In Fig. 4 (bottom), a schematic representation of the used one-dimensional finite element mesh (FE mesh) is shown.

For the time discretization, the backward Euler finite difference method is used

11 $${\dot{c}}_{\mathrm{f}}= {\dot{c}}_{\mathrm{f}}^{n+1 }\approx \frac{{c}_{\mathrm{f}}^{n+1}-{c}_{\mathrm{f}}^{n}}{\Delta t}$$

12 $${\dot{c}}_{\mathrm{b}}= {\dot{c}}_{\mathrm{b}}^{n+1 }\approx \frac{{c}_{\mathrm{b}}^{n+1}-{c}_{\mathrm{b}}^{n}}{\Delta t}$$

where *n* is the actual point in time and *t* is the time. The used time step size is ${t}_{n+1} - {t}_{n} = \Delta t = 0.5 \mathrm{s}$.

### Initial conditions and boundary conditions

The simulation starts right before the slides are placed into the dye basin. Hence, the concentration of the free and of the bound dye are both equal to zero at $t = 0 \mathrm{s}$, i.e., ${c}_{\mathrm{f}}\left(z, t = 0\mathrm{ s}\right)= 0\mathrm{ mol }{\mathrm{m}}^{-3}$ and ${c}_{\mathrm{b}}\left(z, t = 0\mathrm{ s}\right)= 0\mathrm{ mol }{\mathrm{m}}^{-3}$.

At its surface, where the tissue section is in contact with the dye solution, a constant concentration of the free, unbound dye is assumed, ${c}_{\mathrm{f}}$(*z* = 6 µm*, t*) = ${\overline{c}}_{f}$ with ${\overline{c}}_{f}$ being the concentration of the dye used in the respective recipe. At the tissue section base, due to the restricting glass slide, the flux is set to zero, i.e., ${{\varvec{J}}}_{\mathrm{diff}}$(*z* = 0 m*, t*) = 0 s^−1^ m^−2^. In Fig. 4, these two conditions are visualized.

For the bound dye concentration ${c}_{\mathrm{b}}$, no boundary condition is necessary.

### Material parameters

The material parameters used for the comparison and the validation of the model proposed with the experimental data were calibrated from two of five different staining times, with the exceptions of ${I}_{0,i}$ with *i* = *R,G,B*. With the assumption of white light passing through the slide, ${I}_{0,i}$ with *i* = *R,G,B* can be set to ${I}_{0,i}$= 255.

A combination of Merck’s Mayer’s hemalum and SAV’s Mayer’s hemalum with a mixing ratio of 1:2 in favor of SAV’s hemalum recipe was used to stain the tissue sections attached to the slides. The hematin and the mordant form a bond in a ratio of 1:1 (Bettinger and Zimmermann 1991). Therefore, the concentration of the hematin and the same amount of the mordant were summed up. This is done for the recipe of Merck and SAV. From the calculated values, the weighted average is taken. The result is referred to as ${\overline{c}}_{\mathrm{f}}$.

Because no rate constants were found in the literature concerning the binding and the release of hemalum to and from the tissue, two assumptions were made. The first assumption is that the diffusion is the time limiting process (Goldstein 1980; Winzek and Baumgärtel 1988) and the second one that the dye release will be much slower than the binding (Siedel and Zimmermann 1995). For the determination of the rate constants, the inequation

13 $${t}_{\mathrm{binding}}\ll {t}_{\mathrm{diffusion}}\ll {t}_{\mathrm{dye release}}$$

following Fibich et al. (2005) was set up. For the binding time, the diffusion time and the dye release time the following equations were used.

$${t}_{\mathrm{binding}}={\left({k}_{\mathrm{on}}{c}_{\mathrm{b}}^{\mathrm{max}} \right)}^{-1}; \quad {t}_{\mathrm{diffusion}}=\frac{{h}^{2}}{2D}$$

$${t}_{\mathrm{dye release}}={k}_{\mathrm{off}}^{-1}$$

The combination of these equations and the inequation (13) leads to the used inequation

14 $$\frac{{k}_{\mathrm{off}}}{{c}_{\mathrm{b}}^{\mathrm{max}}}\ll \frac{2D}{{h}^{2}{c}_{\mathrm{b}}^{\mathrm{max}}}\ll {k}_{\mathrm{on}}$$

for the determination of the rate constants. In order to achieve that ${k}_{\mathrm{on}}$ is much larger and ${k}_{\mathrm{off}}$ much smaller than $\frac{2D}{{h}^{2}{c}_{\mathrm{b}}^{\mathrm{max}}}$ the resulting values are to be multiplied by a large value. In the present work, $\frac{2D}{{h}^{2}{c}_{\mathrm{b}}^{\mathrm{max}}}$ was multiplied by 500 in order to be physically consistent.

Note that the rate constants are no longer variable parameters but depend on the diffusion coefficient *D*. The parameter ${k}_{\mathrm{on}}$ also depends on the maximum number of binding sites ${c}_{\mathrm{b}}^{\mathrm{max}}$.

The result of staining the nuclei in a histologic section can vary with some nuclei being more intensely stained than others. A possible explanation for this could be the difference in volume of the nuclei. If the number of binding sites per nucleus is the same, a change in volume results in a change in concentration. Another reason which is especially important when dealing with tumors is the fact that tumor cells in comparison to normal parental cells can have a different number of chromosomes or chromosome parts (Haroske et al. 2001). For the staining with hemalum, more chromosomes or more chromosome parts are equivalent to more binding sites and therefore a more intense staining is possible, depending on the used staining time. The amount of dye which each nucleus can bind determines how intense the virtual staining will be. To be able to consider this effect, each nucleus is assigned an individual maximal concentration of free binding sites ${c}_{\mathrm{b}}^{\mathrm{max}}$ for the dye. Therefore, an approximation is needed for ${c}_{\mathrm{b}}^{\mathrm{max}}$. Thus, the assumption was made that the aluminum ions of the hemalum will bind to the phosphor atoms of the cell nuclei (Lang 2006; Prentø 2001; Veuthey et al. 2014). The number of phosphor atoms were then estimated with the following consideration. In a nucleus are two sets of chromosomes. Each chromosome set is built up of 3*.*2 × 10^9^ base pairs. A base pair consists of two nucleotides, which in turn consist, among other things, of a phosphate residue (Rassow et al. 2012). This results in 1*.*28 × 10^10^ phosphor atoms in the DNA per nucleus and therefore 1*.*28 × 10^10^ free binding sites for the hemalum per nucleus. In order to obtain a concentration, the number of particles must be related to a volume. In this paper, the assumption was made that the nucleus has the shape of a sphere and that due to the high aluminum concentration used in the recipe for the hemalum, the dye molecules can bind to all binding sites. For the diameter of the sphere, $d=6 \mathrm{\mu m}$ is chosen, which leads to a concentration of ${c}_{\mathrm{b}}^{\mathrm{max}}$ = 187 mol m^−3^. Please note that the diameters can also be smaller than the thickness of the slide. However, to get a fix point, it was assumed that most of the nuclei have a diameter that is approximately the thickness of the section (Winzek and Baumgärtel 1988). Lang (2006) reported that the aluminum ions can bind to either one or two phosphor atoms. To take this into account, ${c}_{\mathrm{b}}^{\mathrm{max}}$ was divided by the mean value, i.e., 1.5. Since a number of assumptions were made in determining ${c}_{\mathrm{b}}^{\mathrm{max}}$ such as the shape and size of the nuclei or the number of binding sites, a range of values were used for the test calculations (Table 1). Due to the dependency of ${k}_{\mathrm{on}}$ on ${c}_{\mathrm{b}}^{\mathrm{max}}$, ${k}_{\mathrm{on}}$ is also specified through a value range. The diffusion coefficient was fitted using the mean RGB intensities of the slide from the experiments, which was stained for $t = 25 \mathrm{s}$. Please note that the determination of the parameters can also be done at a different time point. However, our preliminary studies have shown that the best results can be obtained at an early time point, i.e., when the diffusion process is not yet completed.

**Table 1:** Material parameters used in the simulation

Parameter	Description	Value	Unit
Binding rate constant	${k}_{\mathrm{on}}$	$0.34\dots 2.52$	${\mathrm{m}}^{3}\mathrm{ mo}{\mathrm{l}}^{-1} {\mathrm{s}}^{-1}$
Dye release constant	${k}_{\mathrm{off}}$	$0.013$	${\mathrm{s}}^{-1}$
Diffusion coefficient	$D$	$4.53\cdot {10}^{-12}$	${\mathrm{m}}^{2}{\mathrm{ s}}^{-1}$
Maximal dye concentration that the tissue can bind	${c}_{\mathrm{b}}^{\mathrm{max}}$	$50\dots 376$	${\mathrm{mol m}}^{-3}$
Dye concentration in the dye basin	${\overline{c} }_{\mathrm{f}}$	$10.67$	${\mathrm{mol m}}^{-3}$
Extinction coefficient hemalum red	${\varepsilon }_{\mathrm{R}}$	$1585$	${\mathrm{m}}^{2}\mathrm{ mo}{\mathrm{l}}^{-1}$
Extinction coefficient hemalum green	${\varepsilon }_{\mathrm{G}}$	$2996$	${\mathrm{m}}^{2}\mathrm{ mo}{\mathrm{l}}^{-1}$
Extinction coefficient hemalum blue	${\varepsilon }_{\mathrm{B}}$	$1111$	${\mathrm{m}}^{2}\mathrm{ mo}{\mathrm{l}}^{-1}$
Intensity of the incoming light red	${I}_{0,\mathrm{R}}$	$255$	${\mathrm{W m}}^{-2}$
Intensity of the incoming light green	${I}_{0,\mathrm{G}}$	$255$	${\mathrm{W m}}^{-2}$
Intensity of the incoming light blue	${I}_{0,\mathrm{B}}$	$255$	${\mathrm{W m}}^{-2}$

The extinction coefficients were fitted using the mean RGB intensities from the experiments for the slide, which was stained for *t* = 100 s. At this time point, the staining is completed (Fig. 6) and therefore, the assumption was made that the concentration of bound dye ${c}_{\mathrm{b}}$ is equivalent to the maximum concentration of free binding sites ${c}_{\mathrm{b}}^{\mathrm{max}}$. Following that, the Beer–Lambert’s law was converted to the extinction coefficients *ε*_*i*_ for *i* = *R,G,B*. With the analytically determined ${c}_{\mathrm{b}}^{\mathrm{max}}$, the thickness of the section *h* and the mean RGB intensities, all unknowns are determined. Of note, the thereby determined values of *ε* are smaller than the values for *ε* according to Bettinger and Zimmermann (1991). They specify a maximal molar extinction coefficient as well as a curve for the extinction over the wavelength for multiple hemalum concentrations with different preparation steps. One explanation for the differences could lie in the fact that the maximal molar extinction coefficient was measured in a solution and not in a tissue section as it was done in the current work.

For the calibration of the material parameters, the data from two staining time points were used. At $t = 25 \mathrm{s},$ the equilibrium was not yet reached and the dynamic parameters could be fitted. In the current work, these parameters are the diffusion coefficient *D* and also to a certain extend the rate constants ${k}_{\mathrm{on}}$ and ${k}_{\mathrm{off}}$ via inequation (14). However, this procedure is only needed because we are not aware of any reliable sources for these parameters. The same applies for the extinction coefficients. If these parameters are all determined through independent experiments, the remaining material parameter that has to be determined is ${c}_{\mathrm{b}}^{\mathrm{max}}$. It is important to perform this with a slide where the equilibrium (end state) of the staining is achieved. If the parameter is fitted at e.g., *t* = 25 s the resulting ${c}_{\mathrm{b}}^{\mathrm{max}}$ would not be the actual maximal concentration of binding sites. This would only make sense if the staining would normally be ceased at this time point because the desired staining has reached saturation. The physical meaning of ${c}_{\mathrm{b}}^{\mathrm{max}}$ would then change from the (total) maximal concentration of binding sites to the maximal concentration of binding sites needed to achieve the desired staining.

The maximal concentration of free binding sites ${c}_{\mathrm{b}}^{\mathrm{max}}$ together with the extinction coefficients *ε*_*i*_ for *i* = *R,G,B* are the parameters, which determine the resulting stain. While the extinction coefficients are the same for the whole slide, ${c}_{\mathrm{b}}^{\mathrm{max}}$ can vary for the nuclei, depending on their size. The material parameters used for the numerical simulation are summarized in Table 1.

### Finite element mesh from a scanned slide

**Fig. 5:**
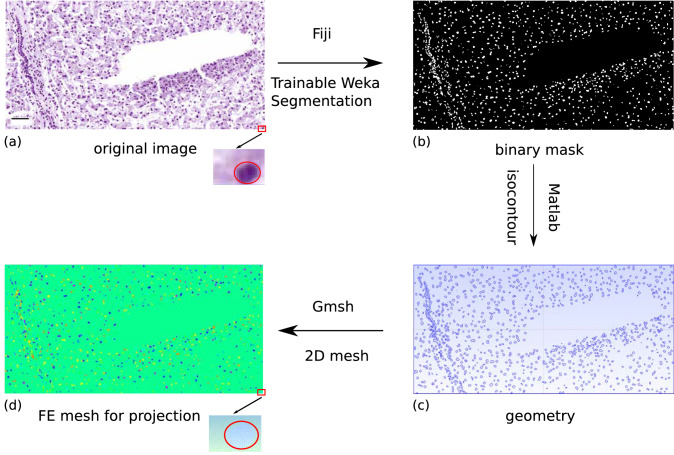
Presentation of the important steps during the creation of an FE mesh based on a section of a scanned histological slide. The slide from which a section is shown in **a** was stained for 13 s with Mayer’s hemalum. In **b**, the binary mask for the cell nuclei is depicted. The geometry is shown in **c**. In **d**, the FE mesh for the projection is presented. Additionally, a single nucleus (*red outlined*) is shown at high magnification. The resulting mesh for this nucleus is shown at the bottom of the image of the “FE mesh for projection”. The* scale bar* is 50 µm

The simulations are conducted on a one-dimensional mesh (Fig. 4). In Fig. 5, the workflow for the creation of a FE mesh based on a real slide is presented. This mesh is used only to record the projected simulation results to create a two-dimensional image (Supplementary Fig. 5). Thus, no computations are carried out with this mesh. In Fig. 5a, a single nucleus is depicted at high magnification. Zooming in on a section of the image shows the nucleus approximated by finite elements (Fig. 5d).

The process starts with a scan of the histological section (Fig. 5a) for which the virtual staining is to be simulated. For the scanning of the slides, the scanner Pannoramic DESK from Sysmex^©^ was used. Afterwards, a section of this image at a magnification of 100 × or 200 × was cut out for further evaluations. This image can then be opened with the program Fiji (Schindelin et al. 2012). The use of a sufficiently high magnification is necessary because otherwise the nuclei cannot be recognized unequivocally. Then, a binary mask was created with the “Trainable Weka Segmentation” plugin, generated by Arganda-Carreras et al. (2017), shown in Fig. 5b. The image is divided into nuclear areas and areas free of nuclei, e.g., cytoplasm or interstitium or void spaces with this mask. The binary mask can now be saved and loaded into MATLAB. With the MATLAB function isocontour (Kroon 2021), the isocontour lines were calculated and the information was saved in form of the isocontour lines and the points that make up those lines (Fig. 5c). For the creation of the actual FE mesh for the projection, the program Gmsh (Geuzaine and Remacle 2009) was used. Therefore, the information of the isocontour lines and points is exported from MATLAB in a syntax, which Gmsh can read. After that, the meshing algorithm from Gmsh can be used to create the FE mesh (Fig. 5d). The different colors are just a visual presentation of (i) the different nuclei (multiple colors) and (ii) the non-nuclear components of the tissue (green).

### Summary of the assumptions

The assumptions made to create the model can be summarized as follows:

1. The concentration of the dye on the surface of the histological slide is constant for the time period under consideration.
2. The reaction of the dye with the tissue is a reversible equilibrium reaction.
3. The diffusion in the *xy*-plane can be neglected.
4. The number of binding sites for the dye in the tissue is fixed ergo there is a ${c}_{\mathrm{b}}^{\mathrm{max}}$.
5. The thickness of the section and the diameter of the cell nuclei inside the histological section are usually in the same range.
6. The cell nucleus has the shape of a sphere.
7. The temperature *T* inside the dye basin is constant.
8. The diffusion coefficient *D* inside the slide is isotropic and homogeneous.
9. There exists only one cell nucleus over the height of the slide.

These assumptions apply only to the staining of liver tissue with hemalum. However, for tissues with similar structure, the basic procedure could be the same. The staining of other tissues or the use of other dyes and staining techniques will be part of future work.

## Results

For validation and comparison of the proposed model, frozen sections of the liver were stained with Mayer’s hemalum for different periods of time. Sections from these slides are shown in Supplementary Fig. 4. Two individual slides were prepared for each of the six staining times$, t = 0 \mathrm{s}, 13 \mathrm{s}, 25 \mathrm{s}, 50 \mathrm{s}, 100 \mathrm{s}, 200 \mathrm{s}$. For simplicity, it was assumed that the staining within a nucleus is evenly distributed. Therefore, it is sufficient to determine the RGB intensities for the central point of the nuclei. For each time, one of the two prepared slides was selected. Three different sections were selected from this slide and investigated at a 200 × magnification to guarantee a sufficient pixel number per nucleus.

Preliminary studies have shown that the magnification should not be too small. With a lower magnification, more cell nuclei can be considered, but as a consequence the number of pixels per nucleus is too low. To counteract this, the 200 × magnification is chosen. Additionally, multiple sections were selected to determine the mean values of the RGB intensities for each staining time point. For these sections, the geometry was approximated and the central point of each cell nuclei be determined. With the coordinates of the central points, the RGB intensities from the sections could be computed.

The binary mask stores the information of whether pixels belong to a nucleus or not. However, this mask may also contain artifacts in which only part of a nucleus was detected or a certain part of the cytoplasm fell into the class of nuclei (Supplementary Fig. 3). In order to filter out incorrectly detected nuclei, the area of each nucleus was computed. Afterwards, only those “nuclei” whose area was larger than *A* = 12(μm)^2^ were processed. The exact value used for the threshold of the area is chosen arbitrarily. The main reason for the need of a threshold value is to filter out small artifacts that are clearly not nuclei (Supplementary Fig. 3).

**Fig. 6:**
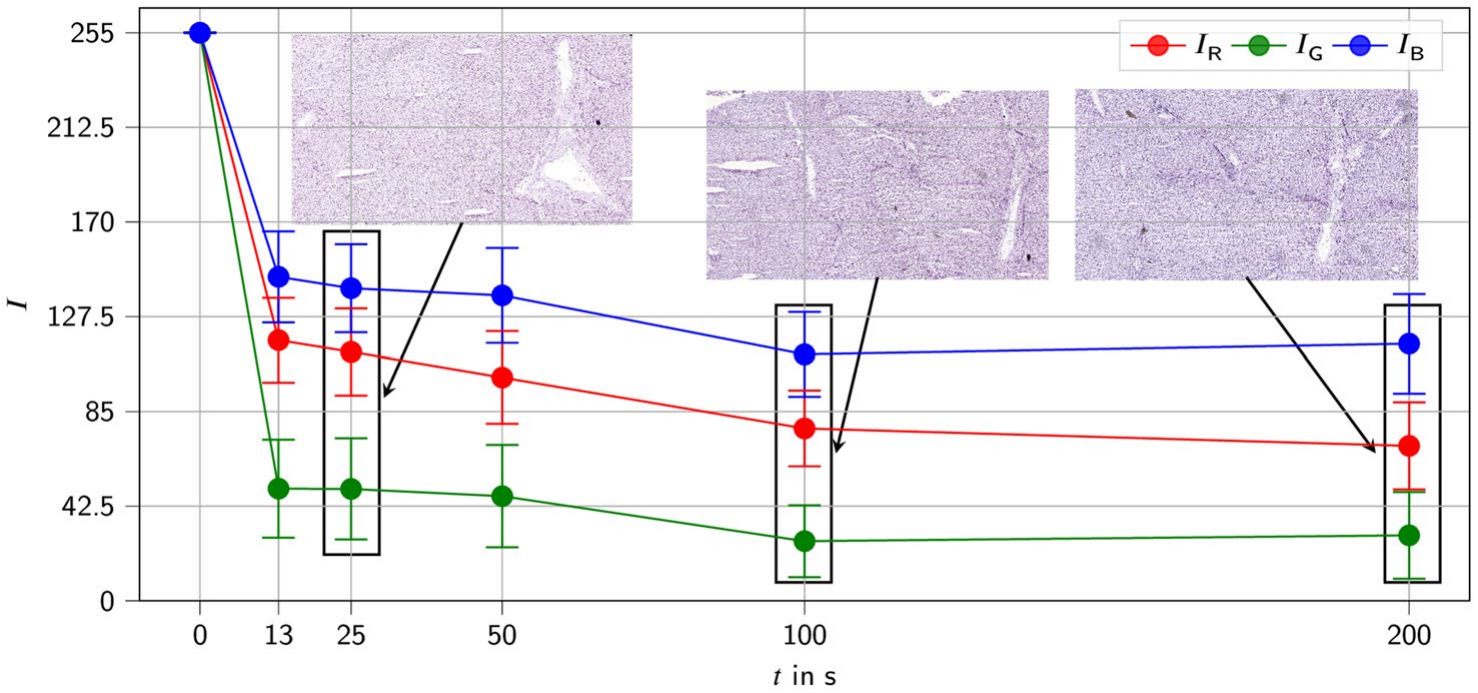
Determination of mean RGB values for different time points of tissue section staining. Three histological slides from which sections were taken are depicted in the image. For each staining time point, a different histological section was evaluated in the experiments

Figure 6 shows the result of combining the two procedures: (i) filtering out the artifacts and (ii) determining the RGB intensities via the central points of the nuclei. Three different sections were evaluated per staining time corresponding to total of approximately 2400 nuclei per staining time.

The error bars defined by the standard deviation are almost the same for each time and quite large. To give an overview of the variation in the RGB intensities that are computed by this method, box plots of the RGB intensities are shown in Supplementary Fig. 6. It can be seen that the number of outliers is quite different for the different sections. However, the number of outliers is small compared to the total values studied. Furthermore, the mean values were fairly similar for all three sections (of the respective histological slide) per staining time.

In Fig. 6 it can be seen that after a staining time of only 13 s $,$ the nuclei already exhibited a very intense staining. For longer staining times, the intensities increased much slower and after $t = 100 \mathrm{s}$ they did not increase anymore (Fig. 6). Therefore, it can be concluded that between $t = 50 \mathrm{s}$ and $t = 100 \mathrm{s}$ an equilibrium has been reached. The mean RGB intensities determined depend on which of the two slides is used for each stain (Supplementary Fig. 2).

The quality of the data can be improved by choosing more similar tissue samples. Another possibility is to remove the same tissue sample at different staining times, which is experimentally demanding. However, it should be noted that this does not impact the simulation results, because only two individual slides are used for the fitting: the first one at $t = 25 \mathrm{s}$ (in disequilibrium) is used for calculating the diffusion coefficient and the rate constants, the second one (in equilibrium) at $t = 100 \mathrm{s}$ is used for the calculation of extinction coefficients as well as the maximal dye concentration, which the tissue can bind.

**Fig. 7:**
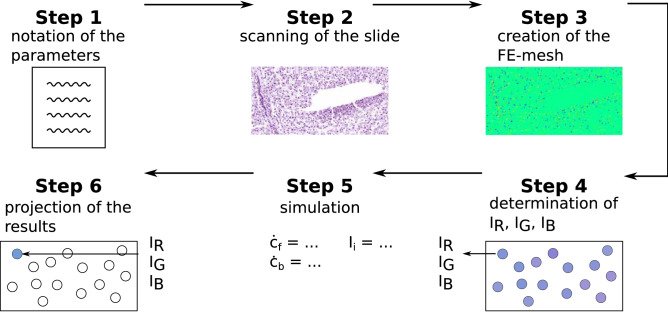
Flowchart of the single steps needed to conduct the simulation for the virtual staining of a histological slide. According to the initial conditions for ${c}_{\mathrm{b}}$ and ${c}_{\mathrm{f}}$, both concentrations are set to 0 mol m^−3^ at $t = 0$ s, cf. Section “Initial conditions and boundary conditions”

The typical workflow for performing a virtual staining is shown in Fig. 7. First, the parameters used for the creation of the histological slide, like the thickness of the tissue section and the dye used, should be written down. After that, the already-stained slide for which the staining with another dye or for another time span should be simulated is scanned. Based on this scanned slide, a FE mesh is created (see “Finite element mesh from a scanned slide”) with which the geometry is determined.

For each nuclei, an individual ${c}_{\mathrm{b}}^{\mathrm{max}}$ is calculated. For that purpose, first the RGB intensities of each nucleus were determined. Subsequently, a series of test calculations were performed with the presented model, in which different values for ${c}_{\mathrm{b}}^{\mathrm{max}}$ were given and the RGB intensities were calculated. This served as a calibration series for the relationship between the local ${c}_{\mathrm{b}}^{\mathrm{max}}$ and the color intensity.

For the determination of the maximal number of binding sites, it is important to use a stained slide. This is also the reason why for this model the starting point is a stained slide and not an unstained one. Another requirement is that the dye with which the physical slide was stained and the dye with which the virtual staining has to be done, have the same binding sites. If a method is found to determine ${c}_{\mathrm{b}}^{\mathrm{max}}$ purely by geometrical features, a virtual staining could also be done on the basis of an unstained slide.

The RGB intensities obtained by the test calculations are then combined with the respective concentration ${c}_{\mathrm{b}}^{\mathrm{max}}$ used as the material parameter to form an interpolation table. This allows the definition of ${c}_{\mathrm{b}}^{\mathrm{max}}$ based on a RGB intensity of a nucleus. Afterwards, the dye concentration can be computed with the Eqs. (2) and (3) and the resulting staining with the Eqs. (6), (7), and (8) for each cell nuclei individually. Finally, the calculated RGB intensities can be projected onto the FE mesh generated in step 3 (Fig. 5). Now, the three intensities are combined to form the respective color. The comparison between numerically simulated and scanned/measured color is shown in Supplementary Fig. 5.

For the validation of the proposed model with the experimental data, the steps described above in the workflow are performed for a tissue section which was stained for *t* = 100 s. With that, the geometry and ${c}_{\mathrm{b}}^{\mathrm{max}}$ are fitted. The distribution of the free and bound dye concentration ${c}_{\mathrm{f}}$ and ${c}_{\mathrm{b}}$ is shown and described in Supplementary Information.

**Fig. 8:**
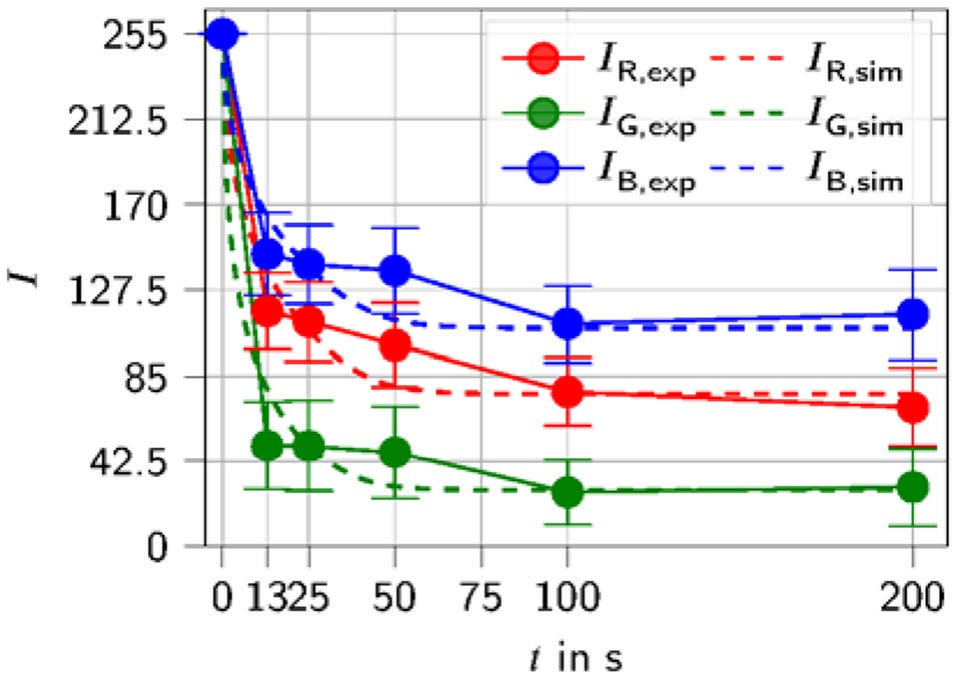
Comparison of the mean values of the RGB intensities of the real, experimental investigated stained slides (exp) with the numerically simulated staining (sim). For the calibration of the geometry and ${c}_{\mathrm{b}}^{\mathrm{max}}$ the used section is depicted in Supplementary Fig. 5

The RGB intensities were calculated for a staining time of 200 s. For the real, physical slides, a different slide was used for each staining time (Supplementary Fig. 4). Therefore, it is not possible to directly compare point-wise (or nucleus-wise) the results of the projected RGB intensities with the real, physical sections. A visual comparison can only be done qualitatively as will be reported below. For a quantitative comparison of the simulation with the experimental results, only a global comparison is possible. The assumption was made that if the global comparison, that is over all the nuclei, is good, the local comparison for the single nuclei would also be good. For a global comparison, the average of the RGB intensities of all nuclei was taken for each simulated second. The simulation results are plotted together with the mean values and error bars of the experimental data versus time in Fig. 8. The simulation results show a fast decrease of the RGB intensities in the first seconds followed by a plateau leading to the final steady-state value (equilibrium value) which is unique for each color. After a staining time of $t = 50$ s the equilibrium seems to be reached. After a staining time of $t = 42 \mathrm{s}$, 95% of the maximal staining is achieved for the simulated data. For $t = 25 \mathrm{s}$ and $t = 100 \mathrm{s}$ the simulation and the experimental data are in excellent agreement. This was to be expected, as the data of the experiments for this time point was used to fit the material parameters used in the equations.

For the other time points investigated, the simulation results were always within the range spanned by the error bars with the exception of ${I}_{G}$ for $t = 13 \mathrm{s}$ and ${I}_{B}$ for $t = 50 \mathrm{s}$. A possible factor for the deviation of the simulation from the experimental results could be the fact that the thickness of the experimental histological sections could be different from the assumed thickness $h = 6 \mathrm{\mu m}$. Please note that $h = 6 \mathrm{\mu m}$ is the height set on the microtome. Since this is a highly precise work step on inhomogeneous (biological) material, variations in the cutting result are quite typical. Estimating the actual section thickness would be one way of adding a correction factor. So far, this has not been possible in standardized applications.

Collectively, our results suggest that the proposed model is well suited to simulate the staining of histological sections. In Supplementary Fig. 6, the box plots of the simulated RGB intensities of the nuclei are depicted. The number of outliers and their value is much lower than for the experimental data. It can be observed that for longer staining times the distribution of the RGB intensities increases.

However, the goal of the proposed model is not to create a diagram as seen in Fig. 8, but to create 2D images of virtually stained histological sections that can be evaluated by an investigator or a machine learning algorithm for cancer detection. In Supplementary Fig. 5, a comparison of simulated staining with the experimental results for the different staining times is shown. It should be noted that only the staining of the nuclei was simulated in the present work. In addition to the stained nuclei (darker purple), the experimental data also include sections of stained cytoplasm (lighter purple), see Fig. 1.

For the staining time of $t = 13 {\mathrm{s}},$ many of the virtually stained nuclei were not as intensely stained as the experimental one (see also Fig. 8). For the later time points, a better agreement between the results of the virtual and the actual staining was found. The variation in intensities of the virtually stained nuclei is larger than in the experimental data (Supplementary Fig. 5). This could be a sign that the process of choosing a ${c}_{\mathrm{b}}^{\mathrm{max}}$ for each nucleus should be improved. Overall, the difference between the simulated staining of the nuclei and the experimental data is within a tolerable range.

## Discussion and outlook

In the present work, a model capable of performing virtual staining of cell nuclei in tissue sections is presented. With the proposed model, we lay the foundation for a histological analysis that can be adapted to the personal habits/experiences of the histologists. It also allows the generation of a much wider variety of training data sets for machine learning methods due to the possibility to standardize the images. Thus, our method allows the application of machine learning-based cancer detection algorithms to slides that are stained with another procedure.

The transport and binding of the dye was computed using a one-dimensional FE simulation. Afterwards, the resulting RGB intensities were determined and the intensities were projected onto a two-dimensional mesh based on a scanned histological slide. When the used material parameters were fitted with the data from two different time points, the obtained curves were generally within the range spanned by the error bars. This means that the model is able to accurately simulate the staining of the nuclei with hemalum over time. In a purely visual comparison, the two-dimensional simulation results are similar to the experimental data. However, there are still small differences, especially for shorter staining times.

With our model, a user can virtually (re-)stain cell nuclei of a histological section from liver tissue, or tissues with similar properties using the same or a different dye. A prerequisite for the applicability of the model is that the cell nuclei of the tissue under investigation form the same bonds with hemalum, which can be assumed at least for most human tissues. For tissue sections of interest, the progress of the RGB intensities at different time points can be visualized. For that, the geometry of the nuclei is approximated. Often a tumor cell is not or not only characterized by a different staining intensity compared to a normal parental cell but also by a larger diameter, which can be detected by the described model. Thus, the investigator or the artificial intelligence model analyzing the virtual staining, perceives this and can make a decision.

An important aspect for further research concerning this topic is the experimental determination of the material parameters like the diffusion coefficient $D$ or the rate constants ${k}_{\mathrm{on}}$ and ${k}_{\mathrm{off}}$. An expansion of the current model, which simulates the staining of nuclei, to include the staining of the cytoplasm is another important aspect. Only the combination of nuclear and cytoplasmic staining will potentially permit a truthful diagnosis.

## Supplementary Information

Below is the link to the electronic supplementary material.

Supplementary file1 (PDF 7428 KB)

## Data Availability

Upon reasonable request.

## Code availability

Upon reasonable request.
