# Supplementary material for: Modeling and simulation of diffusion and reaction processes during the staining of tissue sections on slides
Source: Histochem Cell Biol. 2022 Jun 6;158(2):137–48. doi: 10.1007/s00418-022-02118-9 (PMC9338144; doi:10.1007/s00418-022-02118-9)
Supplement: Supplementary file 1 — Supplementary file1 (PDF 7428 KB) [file 418_2022_2118_MOESM1_ESM.pdf]

Modeling and simulation of diffusion and reaction processes during the staining of tissue sections on slides

Johannes D.M. Menning[1], Thomas Wallmersperger[1,3], Matthias Meinhardt[2*†] and Adrian Ehrenhofer[3,1*†]

[1]Technische Universität Dresden, Institute of Solid Mechanics, George-Bähr-Straße 3c, 01062 Dresden, Germany.
[2]University Hospital Carl Gustav Carus Dresden, Institute of Pathology, Schubertstraße 15, 01307 Dresden, Germany.
[3]Technische Universität Dresden, Dresden Center for Intelligent Materials, School of Engineering Sciences, George-Bähr-Straße 3c, 01062 Dresden, Germany.

*Corresponding author(s). E-mail(s): matthias.meinhardt@uniklinikum-dresden.de; adrian.ehrenhofer@tu-dresden.de;
†These authors contributed equally to this work.

Analysis of the concentration distribution

For the representation of the concentration distributions over the height a single one-dimensional calculation was carried out. The used diffusion coefficient D is the same as in Table 1. For $c_\mathrm{b}^\mathrm{max}$ the mean value of the determined $c_\mathrm{b}^\mathrm{max}$ for the fit of $c_\mathrm{b}^\mathrm{max}$ on a section of the $t = 100\,\mathrm{s}$ stained slide, see the Results section, was used. The resulting maximal concentration and rate constants are $c_\mathrm{b}^\mathrm{max} = 76.8\,\mathrm{m}^3\,\mathrm{mol}^{-1}\,\mathrm{s}^{-1}$, $k_\mathrm{on} = 1.64\,\mathrm{m}^3\,\mathrm{mol}^{-1}\,\mathrm{s}^{-1}$ and $k_\mathrm{off} = 0.013\,\mathrm{s}^{-1}$.

The unbound dye concentration c_f decreases mainly linearly with section thickness, see Supplementary Figure 1. The linear character of this run of the course is a sign that the amount of dye that is bound every second and dye that diffuses into the slide is around the same. With increasing time t the slope of the course for c_f decreases. This is due to the fact, that the top regions of the nucleus have already bound the maximum amount of dye as can be seen in Supplementary Figure 1b. Because of the chosen assumption, see section Material Parameters, the binding of dye occurs faster than the diffusion, see Figure 1b. At the top of the slide, where the height is biggest, the staining is completed. In contrast the lower parts have a much smaller concentration of bound dye and the transition between both parts occurs with a high slope. At $t = 50\,\mathrm{s}$ the nucleus has taken up the maximum concentration of bound dye over the whole height and therefore the staining is completed. This is different compared to the results of the experiments, see Figure 8. Please note that the experimental process is not yet completed after a staining time of $t = 50\,\mathrm{s}$.

Steps towards practical application

In the following, the necessary steps needed for the practical application of the model are summarized.

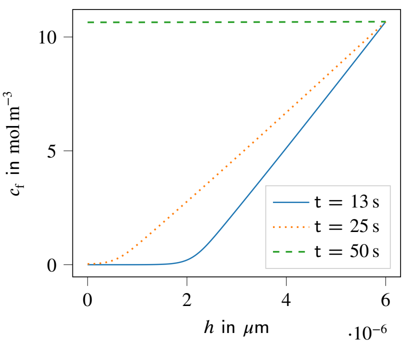

(a) Course of the unbound concentration c_f.

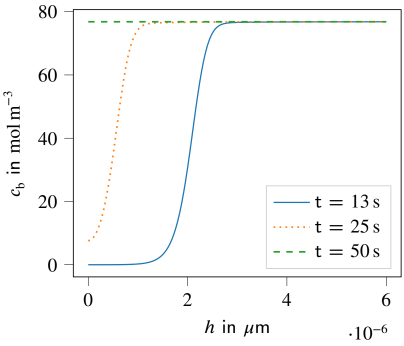

(b) Course of the bound concentration c_b.

Supplementary Figure 1: Representation of the course of (a) the unbound hemalum concentration c_f and of (b) the bound concentration c_b over the height for different staining times, $t = 13$ s, $t = 25$ s and $t = 50$ s. For the computation the equations (2) and (3) and a one-dimensional FE-mesh were used.

- The modeling of at least two chemo-physical stains (for nuclei and cell plasma) is required, so that histopathologists can apply the model to practically relevant staining regimes. This can be further enhanced by adding a third dye (trichrome staining) to distinguish between nuclei, cytoplasm and interstitium.
- A higher geometry model resolution (ideally matching the image resolution) allows the resolution of gradients inside cell nuclei, which is important for pathologists to adequately classify the cell malignancy. - The optimization of the simulation method is required for the implementation of the proposed model into a software tool that allows the real-time restaining of a sample that is placed under the microscope (digital live view or scan).

On the longer perspective, also other mechanisms such as immunohistological staining will be implemented.

Miscellaneous Images

The additional Figures 2 - 6 as explained in the main paper are given in the following.

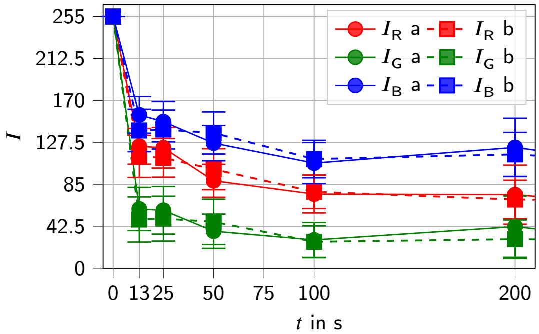

Supplementary Figure 2: In the experiments two slides were stained per staining time. These two slides were labeled a-Series and b-Series. The designation was only used for better clarity and has no further meaning. In the main paper a combination of the results for $t = 25\,\mathrm{s}$ to $t = 200\,\mathrm{s}$ for the b-Series and the values for $t = 13\,\mathrm{s}$ from the a-Series was used. This was done, because it seemed unreasonable that after doubling the staining time from $t = 13\,\mathrm{s}$ to $t = 25\,\mathrm{s}$ no further staining would be seen. The mean RGB intensities are still within the range covered by the error bars for all staining times.

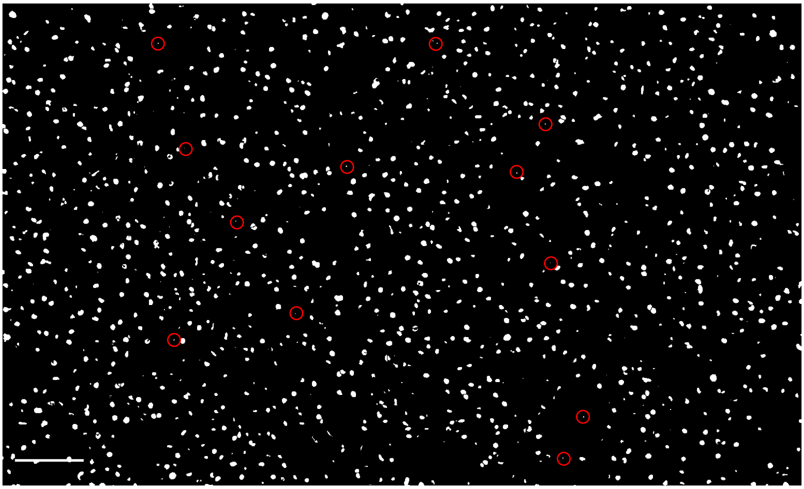

Supplementary Figure 3: Binary mask of a section at a magnification of 200, with some of the components marked as nuclei highlighted by a red circle. Due to their very small size it can be assumed that these are no complete nuclei (but parts that arise when cutting a biological tissue) and are therefore not used in the further computations. The scale bar is 50 µm.

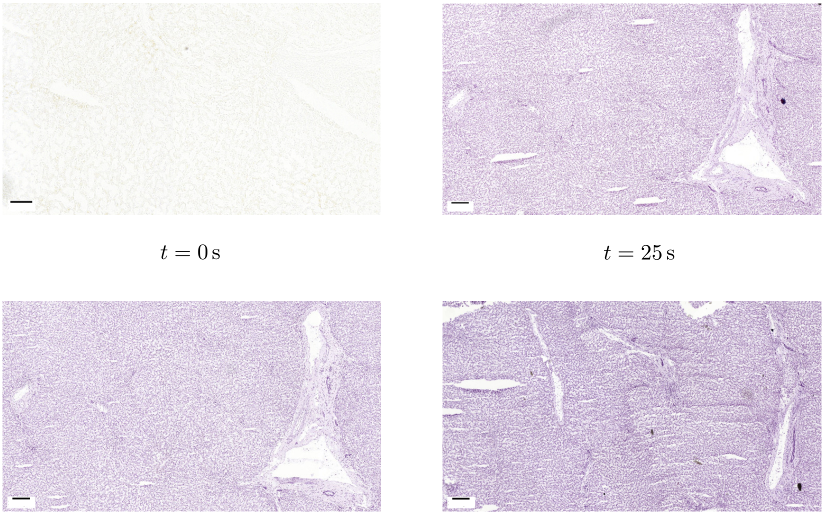

$t = 0\,\mathrm{s}$

$t = 25\,\mathrm{s}$

$t = 50\,\mathrm{s}$

$t = 100\,\mathrm{s}$

Supplementary Figure 4: Representative sections from the histological slides. For every staining time a different slide was created from the processed tissue (liver). The scale bars are 100 µm each.

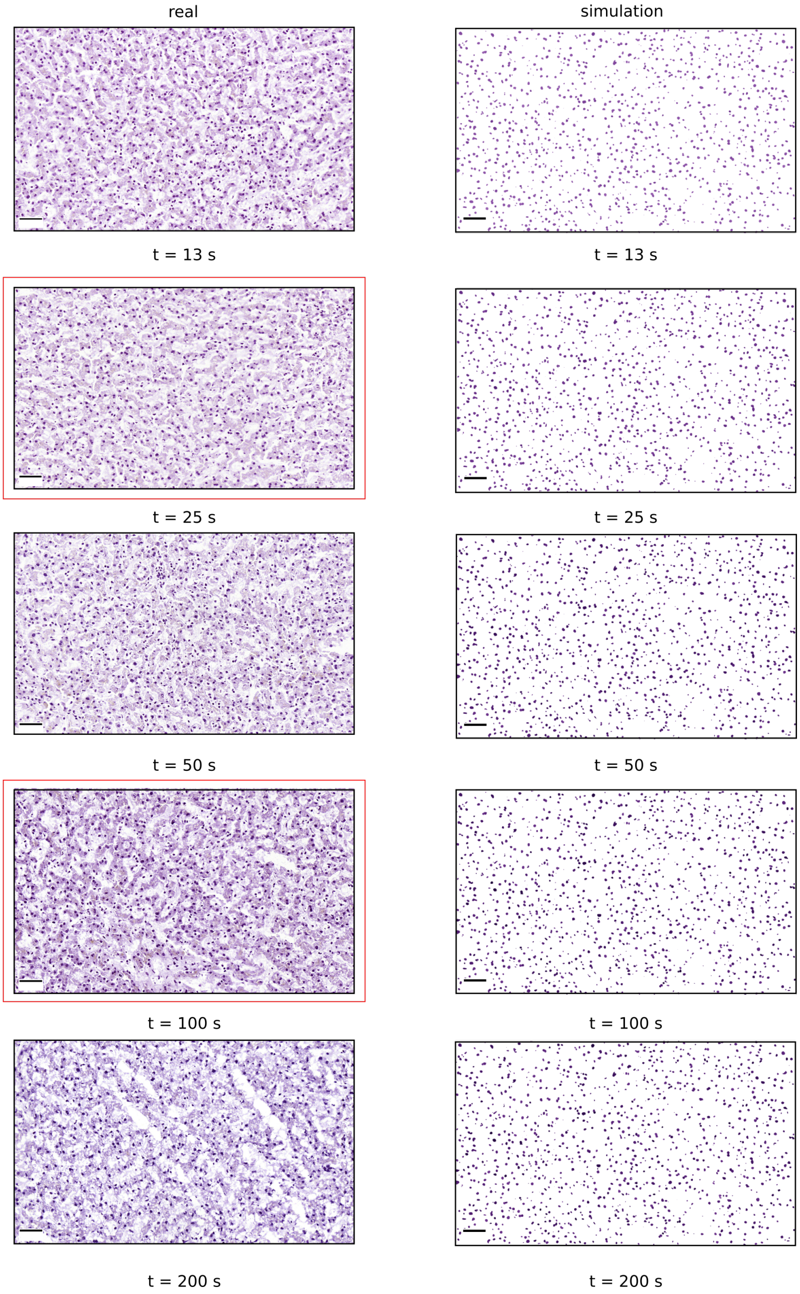

Supplementary Figure 5: Comparison of the real stained sections (left) with the results of the simulated staining (right). The sections used for the calibration of the geometry and the material parameters are highlighted with the red box. The images are from the same sample as those of Figure 4. The scale bars are 50 µm each.

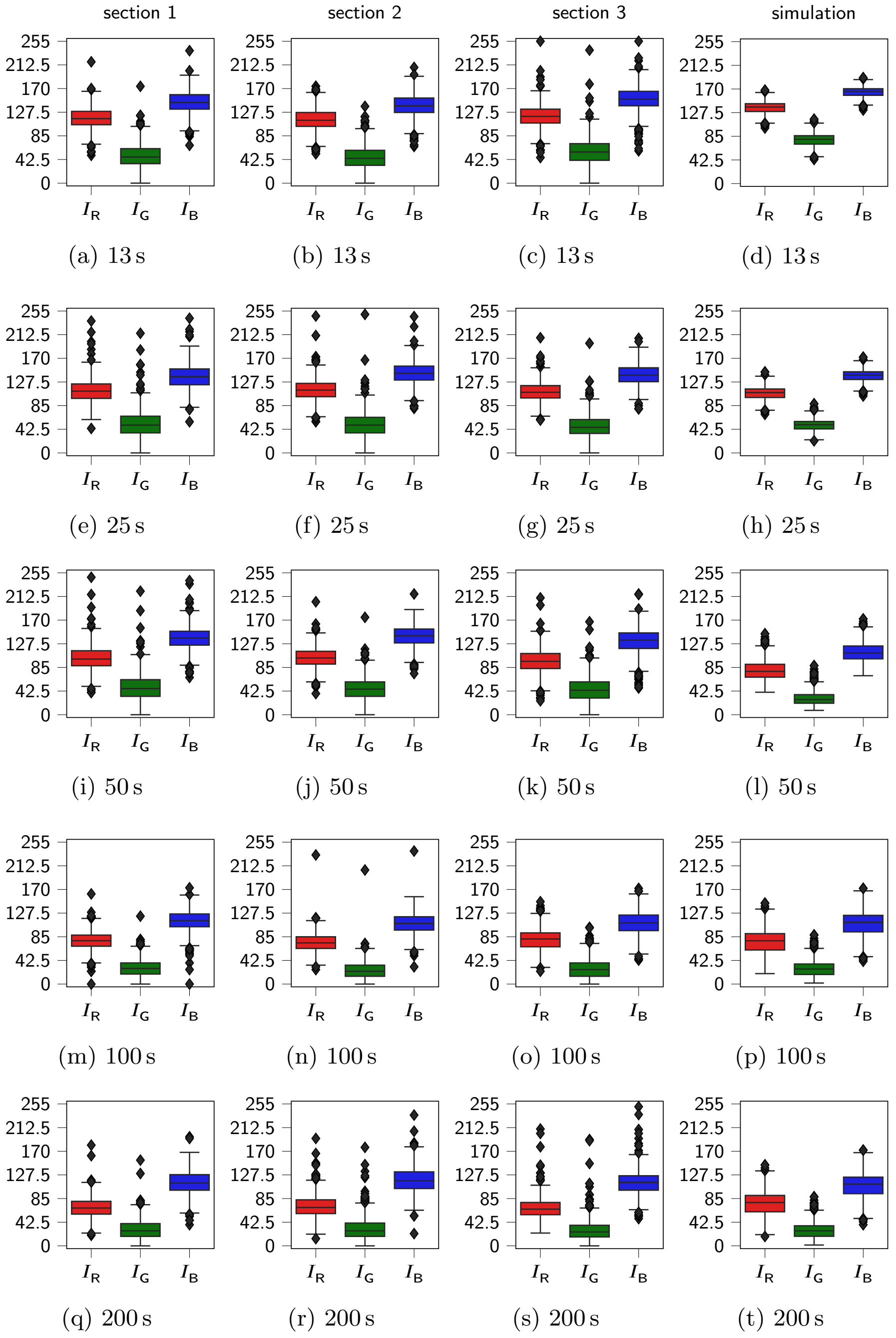

Supplementary Figure 6: Box plots of RGB intensities for the three image section of each experimental histological slide (horizontal alignment) which were stained for a different staining time (vertical alignment). These are the sections which were used to compute the mean values of the RGB intensities. On the right side of each row the box plots from the computed virtual staining are depicted.
